# ^15^N in tree rings as a bio-indicator of changing nitrogen cycling in tropical forests: an evaluation at three sites using two sampling methods

**DOI:** 10.3389/fpls.2015.00229

**Published:** 2015-04-09

**Authors:** Peter van der Sleen, Mart Vlam, Peter Groenendijk, Niels P. R. Anten, Frans Bongers, Sarayudh Bunyavejchewin, Peter Hietz, Thijs L. Pons, Pieter A. Zuidema

**Affiliations:** ^1^Forest Ecology and Management Group, University of WageningenWageningen, Netherlands; ^2^Instituto Boliviano de Investigación ForestalSanta Cruz de la Sierra, Bolivia; ^3^Centre for Crop Systems Analysis, University of WageningenWageningen, Netherlands; ^4^Department of National Parks, Wildlife and Plant ConservationBangkok, Thailand; ^5^Institut für Botanik, University of Natural Resources and Life SciencesVienna, Austria; ^6^Plant Ecophysiology, Institute of Environmental Biology, Utrecht UniversityUtrecht, Netherlands

**Keywords:** stable nitrogen isotopes, nitrogen cycling, anthropogenic nitrogen deposition, tropical forest, tropical tree, tree rings, tree ontogeny

## Abstract

Anthropogenic nitrogen deposition is currently causing a more than twofold increase of reactive nitrogen input over large areas in the tropics. Elevated ^15^N abundance (δ^15^N) in the growth rings of some tropical trees has been hypothesized to reflect an increased leaching of ^15^N-depleted nitrate from the soil, following anthropogenic nitrogen deposition over the last decades. To find further evidence for altered nitrogen cycling in tropical forests, we measured long-term δ^15^N values in trees from Bolivia, Cameroon, and Thailand. We used two different sampling methods. In the first, wood samples were taken in a conventional way: from the pith to the bark across the stem of 28 large trees (the “radial” method). In the second, δ^15^N values were compared across a fixed diameter (the “fixed-diameter” method). We sampled 400 trees that differed widely in size, but measured δ^15^N in the stem around the same diameter (20 cm dbh) in all trees. As a result, the growth rings formed around this diameter differed in age and allowed a comparison of δ^15^N values over time with an explicit control for potential size-effects on δ^15^N values. We found a significant increase of tree-ring δ^15^N across the stem radius of large trees from Bolivia and Cameroon, but no change in tree-ring δ^15^N values over time was found in any of the study sites when controlling for tree size. This suggests that radial trends of δ^15^N values within trees reflect tree ontogeny (size development). However, for the trees from Cameroon and Thailand, a low statistical power in the fixed-diameter method prevents to conclude this with high certainty. For the trees from Bolivia, statistical power in the fixed-diameter method was high, showing that the temporal trend in tree-ring δ^15^N values in the radial method is primarily caused by tree ontogeny and unlikely by a change in nitrogen cycling. We therefore stress to account for tree size before tree-ring δ^15^N values can be properly interpreted.

## Introduction

The rate of natural nitrogen input in tropical forests generally ranges between 2 and 20 kgN ha^−1^ year^−1^, depending on the amount of reactive nitrogen created by lighting and by heterotrophic soil microbes and rhizobia associated with legumes (Vitousek and Sanford, 1986; Galloway, 2004; Pons et al., 2007). Over the last century, nitrogen deposition has strongly increased globally as a result of the widespread use of artificial nitrogen fertilizers and the burning of fossil fuels (Gruber and Galloway, 2008; Davidson, 2009). In the tropics, estimates of anthropogenic N deposition vary greatly, but in large regions N deposition reaches 5–10 kgN ha^−1^ year^−1^, which is about a doubling of natural rates (Galloway et al., 2008; Hietz et al., 2011). The consequences of this increased input are still largely unclear (Galloway et al., 2008). Furthermore, the assessment of the effects of increased N inputs on forests has been limited by a lack of long-term “baseline” biogeochemical data (Gerhart and McLauchlan, 2014). Nitrogen isotopes preserved in wood have the potential to provide these data over a long, multi-decadal to centennial, time scale. There is general agreement that nitrogen isotopes (δ^15^N) in plant material reflect the δ^15^N signature of the available N sources under most field conditions (Evans et al., 1996; Högberg et al., 1999) and thus can provide valuable information on changes in nitrogen cycling in terrestrial ecosystems.

In tropics, the few studies that have measured δ^15^N in tree rings found a consistent increase of δ^15^N during the last decennia in trees from Brazil and Thailand (Hietz et al., 2010, 2011). A similar result was found when comparing historical and current δ^15^N values in leaves from a moist forest in Panama (Hietz et al., 2011). These increases in plant δ^15^N values have been related to higher nitrification and denitrification rates following elevated nitrogen deposition (Hietz et al., 2011; Mayor et al., 2014). In undisturbed tropical rainforest and in the absence of anthropogenic nitrogen deposition, all available mineral N is generally taken up, preferentially as ammonium by trees, leaving little for nitrification (Robertson, 1989; Vernimmen et al., 2007). Increased nitrification will occur when N-availability exceeds N-uptake, which could occur with increasing nitrogen deposition. During nitrification there is a strong fractionation against ^15^N, yielding ^15^N-depleted nitrate (relative to ammonium). If not taken up by vegetation, a fraction of the nitrate will leach downwards and is eventually lost, causing a gradual ^15^N enrichment of the remaining soil nitrogen pool (Högberg and Johannisson, 1993; Högberg, 1997). Denitrification rates can also be increased when N-availability exceeds N-uptake (Corre et al., 2010). A strong discrimination against ^15^N occurs during denitrification (Houlton et al., 2006), leading to the loss of relatively ^15^N-depleted nitrogen oxides and N_2_.

The increases of tree-ring δ^15^N values in tropical trees could thus be evidence for enhanced leaching of nitrate and/or denitrification and suggest tropical nitrogen cycles are becoming more “open” (Högberg, 1990; Högberg and Johannisson, 1993; Hietz et al., 2011). More nitrate leaching can lead to acidification of the soil, which could alter the availability of other nutrients (Matson et al., 1999; Corre et al., 2010). In the long term, such changes can negatively affect plant growth and biodiversity, as is well-known from temperate forests (Magill et al., 2004; Phoenix et al., 2006).

To find further evidence for altered nitrogen cycling (i.e., changing nitrification rates) in tropical forests, we measured δ^15^N values in 400 trees from three sites differing in nitrogen deposition rates. We use a new sampling method that explicitly controls for potential ontogenetic effects by comparing tree-ring δ^15^N values over time across trees with a fixed size (Rozendaal et al., 2010; van der Sleen et al., 2015). We argue that such a control is important, because ontogenetic changes (i.e., during size/age development) could, in theory, also lead to apparent trends in tree-ring δ^15^N values over time. Tree-ring properties (e.g., ring width and stable isotopes) are usually measured in a stem disc from the pith to the bark or, in other words, from the first visible and oldest growth ring up to the last and most recently formed (outer) ring. The period in between may span more than a century, during which changes in tree-ring properties could reflect human-driven environmental changes, like the effects of climate change and increased anthropogenic N depositions. However, during that same period, a tree also grew from a small understorey seedling to a dominant canopy tree. Changes in tree-ring δ^15^N during tree development could result from increased rooting depth with tree size. There is generally a pronounced pattern of ^15^N enrichment in soil profiles, with increasing δ^15^N values with soil depth (Hobbie and Ouimette, 2009). Thus, if trees root deeper when maturing (and forage for nitrogen at greater depths), this may affect the δ^15^N signature of wood in tree rings over time. Alternatively, shifts in the exploited nitrogen sources during tree development can also affect tree-ring δ^15^N values. Different N sources (like NH^+^_4_ and NO^−^_3_) differ strongly in their δ^15^N signature (Hobbie and Högberg, 2012).

Ontogenetic changes could thus potentially obscure, or can be interpreted to reflect, temporal changes in nitrogen cycling and thus need to be accounted for. The new sampling method we apply here is not affected by tree ontogeny because similar sized trees are compared over time. We compare this new sampling method against the conventional method of sampling from the pith to the bark across the stem of large trees. Using both methods, we evaluate evidence for changes in natural ^15^N values in tree rings that could reflect alterations of nitrogen cycling in three tropical forest sites (in Bolivia, Cameroon, and Thailand).

## Materials and methods

### Study area

**Figure 1:**
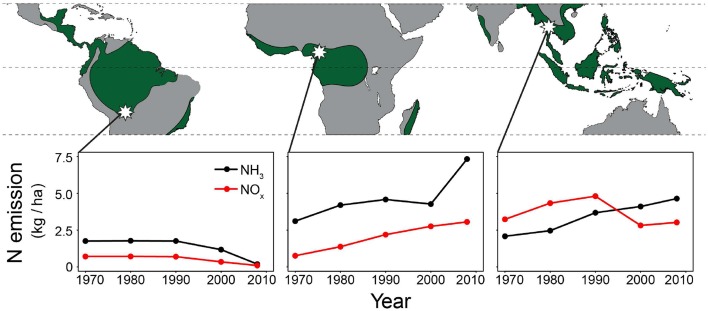
**Study areas (white stars) and anthropogenic NH_3_ and NO_x_ emissions around the study areas (averaged over a 1° grid cell centring the study sites)**. Wood samples were collected from three tropical forests (≥1500 mm rainfall per year; green areas). From left to right: La Chonta logging concession (Bolivia); a logging concession adjacent to Korup National park (Cameroon) and Huai Kha Khaeng Wildlife Sanctuary (Thailand). Nitrogen emission data per 0.1° grid cell are from the European Commission, Joint Research Centre/Netherlands Environmental Assessment Agency, EDGAR version 4.2.

The study was carried out in a forest site in Bolivia (South America), Thailand (Southeast Asia), and Cameroon (Africa; Figure 1). These sites were selected because of the previous work conducted there, which facilitated the collection of samples and provided relatively good background knowledge on these forests (e.g., Groenendijk et al., 2014; Vlam et al., 2014; van der Sleen et al., 2015). Site characteristics are summarized in Table 1.

**Table 1:** **Characteristics of the study sites. See text for details and references**.

**Site, country**	**Av. precipitation mm year^−1^**	**Av. temperature (°C)**	**Soil**	**Soil texture**
La Chonta, Bolivia	1580	24.7	ultisols	sandy-loam
Korup, Cameroon	~4000	26.5	ultisols	sandy
HKK, Thailand	1473	23.5	ultisols	sandy-loam

In Bolivia, trees were sampled in the logging concession “La Chonta,” around 300 km northeast of Santa Cruz de la Sierra (15.84 S, 62.85 W). The forest in La Chonta is a semi-deciduous moist forest and the transitional between Chiquitano dry forest and moist Amazonian forest (Peña-Claros et al., 2008). Annual precipitation in the region averages 1580 mm, with 4–5 months receiving <100 mm from May to September (Peña-Claros et al., 2008). Soils in the study area are mostly derived from gneiss, granitic, and metamorphic rocks and have been described as sandy-loam ultisols (Peña-Claros et al., 2012). They have a neutral pH and a high fertility due to human influences, as ca. 20 percent of the area is being covered by anthropogenic soils, which have a darkened soil with charcoal fragments and pottery shards as evidence of pre-Columbian agriculture (Paz-Rivera and Putz, 2009).

In Thailand, trees were collected in the Huai Kha Khaeng Wildlife Sanctuary (HKK), Uthai Thani province, around 250 km northwest of Bangkok (15.60 N 99.20 E; same study area as Hietz et al., 2011). The vegetation in HKK is a semi-deciduous moist forest (Bunyavejchewin et al., 2009). Mean annual rainfall averages 1473 mm, with a 4–6 months dry season (<100 mm/month) from November to April (Vlam et al., 2014). Soils in HKK are variable, but most are highly weathered ultisols derived from parent material of granite porphyry (Bunyavejchewin et al., 2009). Soil texture is sandy loam at the soil surface, with increasing clay accumulation below 40 cm depth (Bunyavejchewin et al., 2009).

In Cameroon, fieldwork took place in a logging concession (Forest Management Unit 11.001) of Transformation REEF Cameroon (TRC). This area is adjacent to the northwest border of Korup National park, in Western Cameroon (5.23 N, 9.10 E). The forest consists of a semi-deciduous lowland rainforest of the Guineo-Congolian type. Annual precipitation in the region averages around 4000 mm, with 1–3 months receiving <100 mm from December to February (Groenendijk et al., 2014). No detailed information on soil characteristics is available for the area where trees were collected, but the soil in a 50 ha forest plot located ~50 km south of the study site (CTFS Korup plot), is generally skeletal and sandy (up to 70% sand in some areas), with small but increasing clay content with increasing soil depth (Chuyong et al., 2004). Most organic matter is in the top few centimeters of the soil profile and soils are very nutrient poor as a result of the high leaching due to heavy rainfall (Chuyong et al., 2004).

### Nitrogen emissions

Anthropogenic NO_x_ and NH_3_ emission data for the study sites were obtained from the European Commission, Joint Research Centre (JRC)/Netherlands Environmental Assessment Agency (PBL), EDGAR version 4.2 (http://edgar.jrc.ec.europa.eu). Per study site, emission data at a 0.1° grid cell (~11 × 11 km) were averaged over a 1° square (~110 × 110 km) centering the sampled trees. As NO_x_ and NH_3_ can travel through the atmosphere for many kilometers before being rained out, emissions averaged over a 1° grid cell are likely more representative of local nitrogen deposition than at a relatively small 0.1° scale. NH_3_ and NO_x_ emissions at a 1° grid cell were converted to a hectare scale (using the R package SDMTools, which calculates surface areas for spherical polygons based on latitude and longitude coordinates). Results per site are given in Figure 1.

### Study species and collection

At each site, we sampled trees of two species (Table 2). Species were selected based on their abundance (we chose relatively common species) and the possession of clear annual growth rings. The annual nature of growth rings has been demonstrated for the Bolivian species by Lopez et al. (2012), for the species studied in Thailand by Baker et al. (2005), and Cameroon by Groenendijk et al. (2014). At each site, trees were collected in 144–297 ha of undisturbed forest. All trees larger than 20 cm diameter at breast height (dbh) were sampled in a 50 m radius around a randomly assigned gps point. At each site, we used random points spread over the study area and collected around 50 to 100 trees per species (ranging in size from 20 to >100 cm dbh). In Cameroon and Bolivia, a first round of selective logging took place in the study area at the time of sampling (no previous logging had taken place in any of the areas). At these sites, logging operations permitted the collection of stem discs. If no discs could be collected, 5-mm diameter cores were taken using an increment borer (Suunto, Finland and Haglöf, Sweden). Cores were collected in at least three different directions at breast height per tree. After drying, the surface area of discs and cores were either cut or polished depending on what gave the best visibility of ring boundaries.

**Table 2:** **The study species. Species were selected based on their abundance (we chose relatively common species) and the possession of clear annual growth rings**.

**Country**	**Species**	**Family**	**Functional group^a^**	**Sample size**
Bolivia	*Cariniana ianeirensis* *Hura crepitans*	Lecythidaceae Euphorbiaceae	partial shade-tolerant partial shade-tolerant	59 55
Cameroon	*Daniellia ogea* *Terminalia ivorensis*	Fabaceae^b^ Combretaceae	partial shade-tolerant long-lived pioneer	91 84
Thailand	*Melia azedarach* *Toona ciliata*	Meliaceae Meliaceae	long-lived pioneer long-lived pioneer	63 49

a *Functional groups are based on the definitions in Poorter et al. (2006)*.

b *Non-nodulating (Diabate et al., 2005)*.

### Tree-ring identification and sampling strategy

Growth rings were identified using a LINTAB 6 measuring table or using high-resolution scans (1600 dpi) and WinDendro software (Regent Instruments, Canada). Rings were identified for each tree in at least three different directions following standard dendrochronological approaches (Stokes and Smiley, 1996). For each tree, we visually cross-dated (i.e., matched) the ring-width series from three different directions. Matching the ring-width series within the same tree allows the detection of locally absent (missing) or false rings.

**Figure 2:**
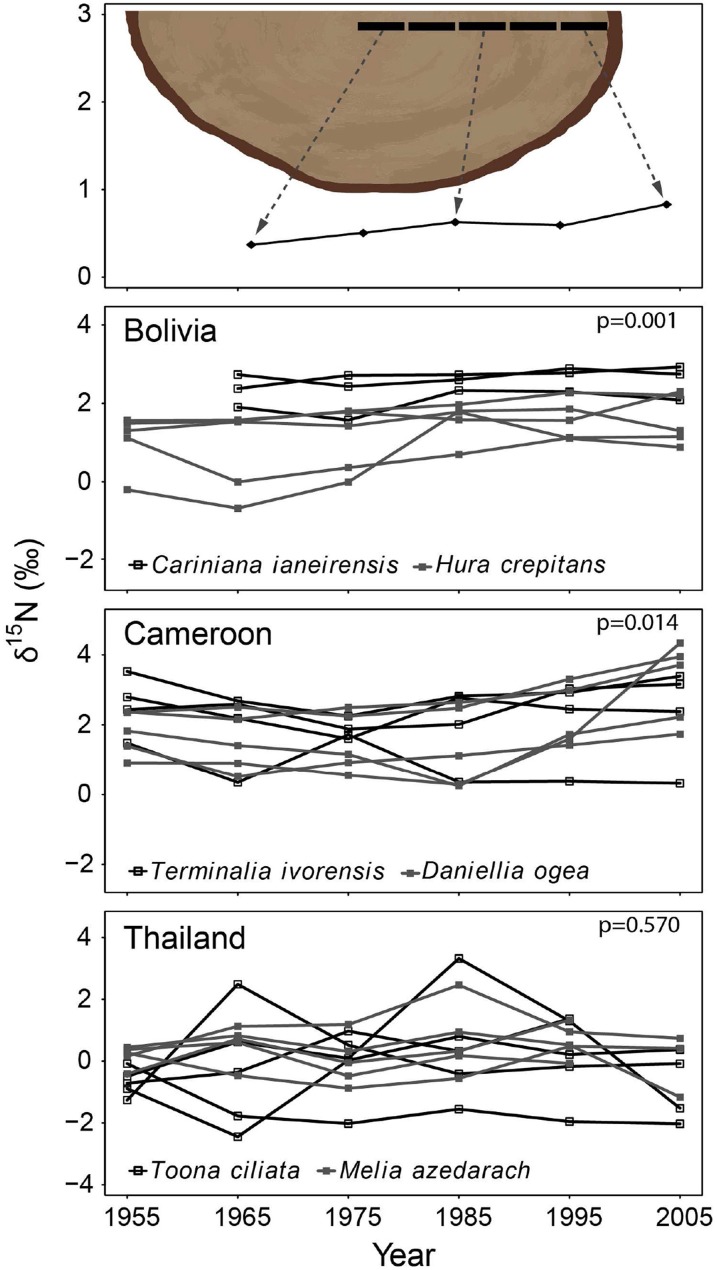
**Tree-ring δ^15^N-values in time using the method illustrated in the top panel (10-year bulk wood samples taken radially from 1950 to 2010)**. For each site, the two species were combined in a mixed-effect model, including “calendar year” as a fixed factor and “individual tree” as a random factor. A significant increase of tree-ring δ^15^N-values over time was found in the trees from Bolivia and Cameroon, but not in trees from Thailand (*p*-values of mixed-effect models in each panel; full results in **Table 3A**).

We collected wood samples with two different methods. In the first, 10-year wood samples over the period 1950–2010 were collected in 28 large trees (five trees per species, except of only three trees in *Cariniana ianeirensis*). For each tree, these wood samples were taken radially (Figure 2 top panel), that is from pith (i.e., the most inner and oldest growth ring) to bark (i.e., the most outer and recent formed growth ring). We only focused on 1950–2010 because this is the period during which changes in nitrogen cycling due to anthropogenic nitrogen depositions might have occurred. We will refer to this method as the “radial” sampling method.

**Figure 3:**
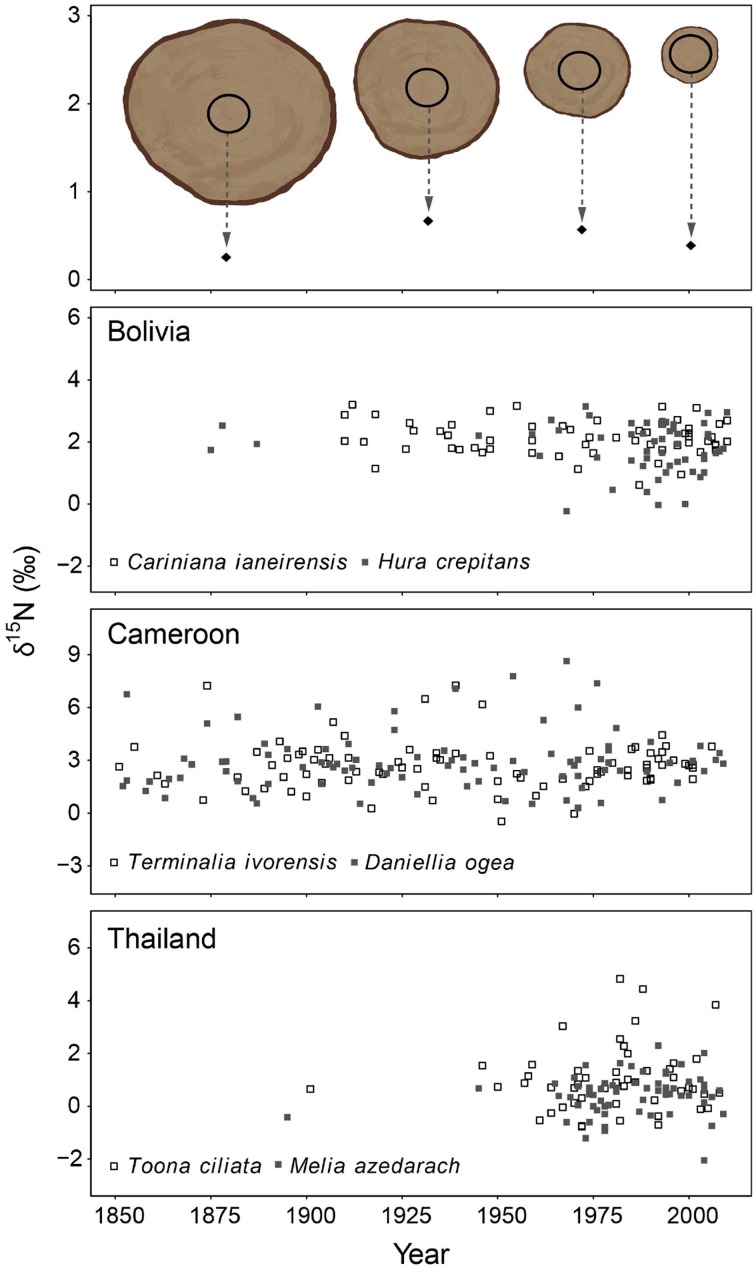
**Tree-ring δ^15^N-values in time using the fixed-diameter method illustrated in the top panel (10-year bulk wood samples around 20 cm dbh)**. Each point in the graph thus represents an individual tree sampled around the same size. Differences in the size of sampled trees allowed a comparison of δ^15^N-values in similar sized trees over time. For each site, the two species were combined in a mixed-effect model, including “calendar year” as a fixed factor and “tree species” as a random factor. No significant change of tree-ring δ^15^N-values was found in Bolivia and Cameroon (*P* > 0.05). In Thailand, a trend of increasing tree-ring δ^15^N-values was found over the period 1950–1990 only (estimate of year effect: 0.0272 ± 0.016, *p* = 0.086). Results of mixed-effect model analyses in **Table 3B**.

In the second method, we control for tree ontogeny by comparing δ^15^N values over time across similar sized trees. In this method, 10-year bulk wood samples were collected at a fixed diameter (see illustrated in Figure 3 top panel). The diameter used was 20 cm dbh and was chosen because trees with a diameter of 20 cm are relatively large (with crowns in the sub-canopy) and likely possess well-developed root systems. Much larger diameters would require the sampling of very large trees to obtain information of the distant past (Figure 2 top panel). For each tree, we sampled the ring formed when the tree reached 20 cm (the “central” ring), as well as the four rings formed before and the five rings formed after the central ring. This yielded a bulk sample of 10 growth years for each tree around a diameter of 20 cm. A total of 405 trees were sampled in this way. Because we collected trees ranging in size from 25 to >100 cm dbh, the rings formed around the 20 cm diameter differed in age. This allowed an analysis of tree-ring δ^15^N values over time across trees in the same ontogenetic stage (Figure 3 top panel). We will refer to this sampling method as the “fixed-diameter” sampling method.

### Stable nitrogen isotopes

The 10-year bulk samples of coarse wood were ground until a very fine powder was formed using a mixer mill (Retsch MM301, Germany). Soluble nitrogen compounds were extracted from the wood samples following Saurer et al. (2004) and Hietz et al. (2010). This extraction removes most of the labile nitrogen and can improve isotope signals (Elhani et al., 2003). Between 20 and 50 mg of ground wood was placed in 2 ml vials. We subsequently added a 1 ml of toluene/ethanol (1:1) for 4 h, followed by 1 mL of ethanol for 4 h, and finally de-ionized water for 1 h. The entire extraction was performed at 50°C. Between extractions and after rinsing with water, the samples were centrifuged at 10,000 rcf for 5 min, the supernatant discarded and the wood samples oven-dried at 60°C for 48 h.

Wood δ^15^N values and nitrogen content (in %) were measured on 10 ± 1 mg of each sample at the Department of Chemical Ecology and Ecosystem Research, University of Vienna, with an elemental analyzer (EA 1110, CE Instruments, Milan, Italy) operating in continuous-flow mode and coupled through a ConFlo III interface (Finnigan MAT, Bremen, Germany) to a gas isotope ratio mass spectrometer (DeltaPLUS, Finnigan MAT). Tree-ring δ^15^N values are expressed relative to the δ^15^N of atmospheric N_2_. The standard deviation of the repeated measurement of δ^15^N in standard material was 0.27‰.

### Statistical analyses

Differences between species were analyzed by comparing the average δ^15^N over the period 1950–2000 between the two species per site in a *t*-test. Subsequently, the site-specific δ^15^N values (for 1950–2000) were compared across the three sites using a One-Way ANOVA and a Bonferroni *Post-hoc* test.

Linear mixed-effect models were used to assess the presence of temporal trends in tree-ring δ^15^N. For the data obtained in the radial sampling method, the two species per site were analyzed in a mixed-effect model that included “calendar year” as a fixed factor. “Individual tree” was included as a random factor to account for the repeated measurement structure of the data. For the fixed-diameter method, we analyzed the data of the two species per site with a mixed-effect model that included “calendar year” as a fixed factor and “tree species” as a random factor.

We estimated the statistical power of the mixed-effect model employed on the data from the fixed-diameter method with a power test. To this end, we simulated data based on the observed data and forced in different temporal δ^15^N trends. For each species a simulated dataset was created using the de-trended variance in δ^15^N in the observed data of that species. The mean δ^15^N value of a species was taken as the values at time = 0. For example, for *Cariniana ianeirensis* the mean δ^15^N value over the period 1900–2011 is 2.13‰, the residual standard deviation is 0.55‰. We created data closely resembling the observed data by randomly adding (following a normal distribution with mean = 0 and standard deviation = 1) the residual standard deviation of 0.55 to the mean of 2.13. This was done for each x-axis value in the observed data, so that the simulated data had the same sample size and same range on the x-axis. A temporal δ^15^N trend since 1950 was inserted by adding a linear increase to the simulated data in time. This was done for each species separately. The simulated datasets of the two species per site were subsequently combined. Per site, we tested 20 trends ranging from a total increase since 1950 from 0.1 to 2‰. For each trend, we generated 1000 datasets and for each of these datasets we tested if a mixed-effect model identical to the one used for the observed data detected a significant effect of “calendar year.” The number of cases for which “calendar year” was significant was divided by 1000 to obtain the estimated power of the model and data to detect a given long-term change in δ^15^N.

All analyses were performed in R, version 2.12.2, (R foundation for Statistical Computing, Vienna, Austria), using the package nlme.

## Results

### Species and site differences in δ^15^N

**Figure 4:**
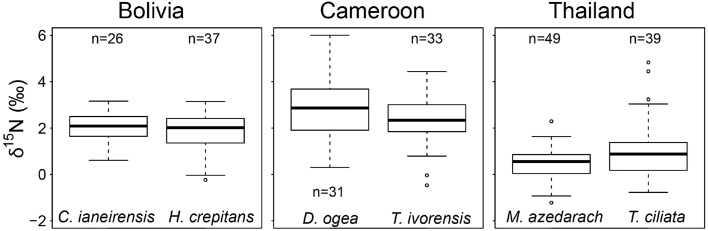
**Average δ^15^N-values from 1950 to 2000 per species**. Data from the fixed-diameter method were compared (Figure 3). Per site, average δ^15^N values of the two species studied were similar, except for Thailand (*p* = 0.023). When δ^15^N values of the two species per site were averaged, δ^15^N was higher in Cameroon compared to Bolivia (*p* < 0.001) and Thailand (*p* < 0.001) and average δ^15^N-values were lower in Thailand than in Bolivia (*p* < 0.001).

We averaged tree-ring δ^15^N values from similar sized tree over the period 1950–2010 per species (data of fixed-diameter method in Figure 3). We found no significant difference in average tree-ring δ^15^N values between the two species from Bolivia or between the two species from Cameroon (Figure 4). In Thailand, average δ^15^N values were significantly higher in *T. ciliata* than in *M. azedarach* (*t* = 2.34, *p* = 0.023). Average δ^15^N values were significantly different between sites (combining the two species per site over the period 1950–2000; *F* = 104.05, *p* < 0.001). The δ^15^N values were higher in Cameroon compared to Bolivia (*p* < 0.001) and Thailand (*p* < 0.001) and average δ^15^N values were lower in Thailand than in Bolivia (*p* < 0.001; Figure 4).

### Radial trends in δ^15^N

The δ^15^N values measured from 1950 to 2010 in 28 large tree species gradually increased in most individuals, but there was a high variance and several trees also showed decreased or constant δ^15^N values over time (Figure 2). When we combined trees in a mixed-effect model (including “individual tree” as a random factor), we found a significant increase of δ^15^N values over the period 1950–2010 in the trees from Bolivia (*p* = 0.0014; Table 3A). This increase was 11.2% per decade, leading to a total increase in δ^15^N of 0.85‰ over the period 1950 to 2010. For the trees in Cameroon, we also found a significant increase of δ^15^N values over the period 1950–2010 (*p* = 0.014; 8.7% increase per decade), amounting to a total increase of 0.84‰ since 1950. No significant change of δ^15^N values was found in the trees from Thailand (Figure 2; Table 3A).

**Table 3:** **Linear mixed-effect model results on temporal changes in δ^15^N values of trees from three sites. The analyses were performed on the data from two sampling methods. (A) The radial method (Figure 2 top panel). For each site “calendar year” was included as a fixed factor, “individual tree” as a random factor in the mixed-effect model analysis. (B) The fixed-diameter method (Figure 3 top panel). For each site, “calendar year” was included as a fixed factor, “tree species” as a random factor**.

	**Period analyzed**	**Estimate of year**	***SE***	***df***	***p*-value**
**(A) RADIAL SAMPLING METHOD**					
Bolivia	1950–2010	0.0142	0.0041	36	0.001
Cameroon	1950–2010	0.0140	0.0055	44	0.014
Thailand	1950–2010	0.0061	0.0052	46	0.570
**(B) FIXED-DIAMETER METHOD**					
Bolivia	1870–2010	−0.0025	0.0021	111	0.230
Cameroon	1850–2010	0.0010	0.0027	172	0.692
Thailand	1890–2010	0.0061	0.0052	109	0.251

### δ^15^N trends in similar size trees

In the fixed-diameter method, we analyzed if trends in tree-ring δ^15^N values were present after controlling for tree ontogeny. Per site, both species were analyzed together in a linear mixed effect model (including “tree species” as a random factor). For the 114 trees from Bolivia, we found no significant change of δ^15^N over the period 1875 to 2005 (Figure 3; Table 3B). Similarly no significant trend was found in the 175 trees from Cameroon from 1851 to 2005, or in the 112 trees from Thailand from 1895 to 2005 (Figure 3; Table 3B).

### Nitrogen content in tree rings

The nitrogen content (as %N of sample dry weight) was determined for all samples. It has become clear however, from numerous studies assessing wood [N] patterns, that the primary drivers of tree-ring [N] are physiological in nature and do not reflect ecosystem N availability (Gerhart and McLauchlan, 2014). As such, we did not use the [N] results for our methodological comparison or for the assessment of the potential effects of anthropogenic nitrogen deposition. We found that nitrogen content in tree rings followed the pattern that is generally found, with highest nitrogen content in the latest year of growth and concentrations decreasing with increasing age. This pattern was apparent in all species and for both methods (Figures S1, S2).

### Statistical power

**Figure 5:**
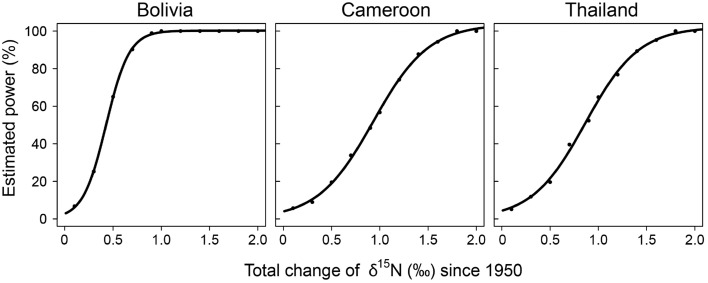
**Statistical power of the linear mixed-effect models used to detect long-term changes in δ^15^N in the fixed-diameter sampling method**. For each change in δ^15^N (x-axis), 1000 datasets were simulated, based on the actual variance in the observed data. For each data set we tested if a mixed-effect model identical to the one used on the observed data detected a significant effect of “calendar year.” The number of cases for which “calendar year” was significant was divided by 1000 to obtain the estimated power of the model (y-axis).

To assess the possibility that the lack of finding significant trends in δ^15^N values over time with the fixed-diameter method (Figure 3) was caused by a limited sample size, we estimated the statistical power of the employed linear mixed-effect models. We tested the power to detect changes over the period 1950–2005. The simulated changes ranged from a total increase of δ^15^N values of 0.1 to 2‰ since 1950. The increase of δ^15^N values of 0.85‰ since 1950 found radially in trees from Bolivia would have been detected with a 99.4% certainty in the fixed-diameter method (Figure 5). For the other two study sites, the power to detect changes was much lower. For trees from Cameroon, the 0.84‰ increase of δ^15^N values from 1950 to 2010 found radially, would have been detected with a probability of only 54.5%. For all sites, the statistical power to detect a total increase (or decrease) of 1.5‰ since 1950 would have been detected with a 90–99% probability (Figure 5).

## Discussion

### Evaluating evidence for changed nitrogen cycling

We used two different sampling methods to evaluate if tree-ring δ^15^N values in trees from tropical forests have consistently changed over the last decades. Such changes could indicate altered nitrogen cycling. We will discuss the results per study site.

For the sampled trees from Bolivia, no change in tree-ring δ^15^N values was found over time (Figure 3; Table 3B), even though the statistical power was high to detect relatively small changes (Figure 5). Constant δ^15^N values however, agree with our expectation for this location, because anthropogenic nitrogen deposition is likely still low in the area (Figure 1). Surprisingly, a significant increase of tree-ring δ^15^N values was found when analyzing δ^15^N within 8 large trees (*p* = 0.0014), which amounted to a total increase of δ^15^N values by 0.85‰ from 1950 to 2010 (Figure 2; Table 3A). If this trend reflects an effect of anthropogenic nitrogen deposition (as found in some tropical forests, e.g., Hietz et al., 2011), we should have detected it with a 99.4% certainty in the fixed-diameter method (Figure 5). The lack of any significant change found when using the fixed-diameter method therefore suggests that this increase is likely due to tree ontogeny, because it disappears when controlling for tree size. These results make an important case for the necessity to control for tree size when interpreting trends in tree-ring δ^15^N values.

For the study site in Cameroon, no significant change in tree-ring δ^15^N values over time was found using the fixed-diameter method (Figure 3; Table 3B). However, a significant increase was found when analyzing δ^15^N within 10 large trees from the same site (*p* = 0.014; Figure 2). This again seems to point to an effect of tree ontogeny, rather than to changes in nitrogen cycling. But we also found that our power to detect temporal changes in δ^15^N was very low in Cameroon, even though we included 175 trees in the fixed-diameter method. Our statistical power to detect the 0.84‰ increase since 1950 found radially (Figure 2) was only 54.5%, which makes it difficult to determine with a high certainty that the found trend within the large trees is solely caused by tree ontogeny. In addition, for the study site in Cameroon, we expected to find an affect of anthropogenic nitrogen input, because reconstructed NH_3_ and NO_x_ emissions are relatively high in the area (Figure 1).

In addition to a low statistical power, we explore two other possible reasons for not finding a change in tree-ring δ^15^N over time. The first relates to natural nitrogen availability. In nitrogen-limited forests, increased anthropogenic nitrogen input might not directly lead to a ^15^N enrichment of the soil nitrogen pool, because all mineral nitrogen will be readily absorbed by the vegetation, leaving little for nitrification and leaching of ^15^N-depleted nitrate (Macdonald et al., 2002). There is very limited information on the nitrogen availability in the forest site in Cameroon, but nitrogen-poor sites have been repeatedly differentiated from nitrogen-rich sites by more negative δ^15^N values (Garten, 1993; Garten and Van Miegroet, 1994; Pardo et al., 2002; Koba et al., 2003). The relatively high average δ^15^N values in the trees from Cameroon compared to Bolivia and Thailand (Figure 4), suggest the opposite case: a forest that might not be strongly nitrogen limited.

Another possible reason for the absence of a long-term trend in δ^15^N values in the Cameroonian forest is that actual nitrogen input was much lower than assumed from the estimated NH_3_ and NO_x_ emissions (Figure 1). Obviously, emissions are not the same as depositions. Emitted NO_x_ and NH_3_ can travel through the atmosphere for many kilometers before raining out. The main components of the estimated nitrogen emission in the region are forest and agricultural land burning. Fires can cause large nitrogen emissions (Palacios-Orueta et al., 2005) and boost estimates of average regional nitrogen emissions. However, the very low population density in a 100 km radius around the study site suggests that nitrogen emissions by fire are likely localized and occur over a short period of time. Thus, nitrogen deposition at the site where trees were sampled may be lower than expected from estimated regional NO_x_ and NH_3_ emissions.

For the trees from Thailand, no significant change of δ^15^N values in tree rings was found in either method (Figures 2, 3). This is a surprising result, because pervious research using the same tree species at the same location found a highly significant increase of tree-ring δ^15^N values over time (Hietz et al., 2011). Although Hietz et al. (2011) analyzed trees radially, they accounted for tree ontogeny, at least partially, by including tree size as a covariate. The discrepancy between these studies could have been caused by a low number of trees included in the radial method of our study (10 vs. 68 trees by Hietz et al., 2011). Given the relatively large variability of δ^15^N values in trees from Thailand (Figure 2), it is possible that we were not able to detect an increased tree-ring δ^15^N when only analyzing 10 trees. The same is likely true for the fixed-diameter method (Figure 3). Although we sampled 112 trees, our power test revealed a low statistical power (64%) to detect the ca. 1‰ increase since 1950 found by Hietz et al. (2011). This makes it possible that a temporal change in tree-ring δ^15^N went undetected in our study.

Hietz et al. (2011) show the strongest increase of δ^15^N over the period 1950–1990 in *Toona ciliata* and a slight decrease after 1990. For *Melia azedarach* a slight increase from 1960 to 1990 was found, but tree-ring δ^15^N values remained more or less constant after 1990 (Hietz et al., 2011). When we only analyzed the period 1950–1990 in the fixed-diameter method, we found some evidence for a trend in tree-ring δ^15^N over time (estimate of year effect = 0.0272, *SE* = 0.016, *p* = 0.086). This result supports the presence of a change in tree-ring δ^15^N values that cannot be related to a size or age effect. It is also unlikely that such a change is caused by human disturbances (e.g., logging), as the study site is a remote and well-protected forest. Because anthropogenic nitrogen emissions are high in the region (Figure 1), the increase of tree-ring δ^15^N values over the last decades found by Hietz et al. (2011) and partially in our study, could reflect the effects of anthropogenic nitrogen deposition. Increased nitrogen input can increase leaching of ^15^N-depleted nitrate (Macdonald et al., 2002), leading to an enrichment of ^15^N in the remaining soil nitrogen pool and subsequently of nitrogen in tree-rings. In the long term, increased nitrate leaching could lead to soil acidification (Vitousek et al., 1997) and negatively affect tree growth by an increased leaching of other essential nutrients with higher soil acidity (Schulze, 1989; Aber et al., 1998; Magill et al., 2004).

### A new sampling design: advantages and disadvantages

Assessing the effect of anthropogenic nitrogen input on nitrogen cycling in tropical forests using the natural abundance of ^15^N in tree rings is not straightforward. Our new methodology accounted for potential ontogenetic effects by comparing tree-ring δ^15^N values in time across similar sized trees (Figure 3 top panel). When δ^15^N is measured within the same tree radially (i.e., from pith to bark; Figure 2 top panel), changes in δ^15^N associated with an altered N cycle can be confounded by ontogenetic changes. However, the presence of ontogenetic changes in tree-ring δ^15^N values still lacks empirical support, although Hietz et al. (2010) found a significant increase of tree-ring δ^15^N with tree age. For wood nitrogen content, it is commonly found that [N] is highest in the latest growth ring and decreases with tree-ring age (e.g., Poulson et al., 1995; Choi et al., 2007). We also found decreasing nitrogen content from bark to pith in most trees and using both methods (Figures S1, S2). These trends have been mainly related to physiological processes within the tree (i.e., the re-use of nitrogen present in cambial cells during maturation) and not to environmental factors (Gerhart and McLauchlan, 2014). Interestingly, we did not find a consistent correlation between tree-ring δ^15^N and nitrogen content (results not shown). This was especially notable for the data from the radial-sampling method, in which increases in δ^15^N were observed. It thus seems that other factors than plant physiology underlie the observed trends in δ^15^N within trees (results Figure 2). We will briefly discuss two possible factors that could lead to shifts in tree-ring δ^15^N values during tree development; their presence however lacks empirical evidence.

Firstly, plants absorb most nitrogen in the form of either ammonium or nitrate (Högberg, 1997). Ammonium and nitrate differ in δ^15^N, with nitrate usually depleted in ^15^N compared to ammonium (Mariotti et al., 1981; Nadelhoffer and Fry, 1994; Robinson, 2001). Thus any shift in the uptake of ammonium relative to nitrate during tree development will lead to a change in tree-ring δ^15^N values.

Secondly, ontogenetic changes in δ^15^N could be the result of increased rooting depth with size. δ^15^N often increases with soil depth, with the highest δ^15^N values at intermediate depth in less nitrogen-limited forests (Hobbie and Ouimette, 2009). These profiles are the result of both ^15^N-depleted plant litter at the soil surface and the loss of ^15^N-depleted nitrogen during denitrification at intermediate depths where temporary anoxic conditions favor denitrification (Hobbie and Ouimette, 2009). Increasing rooting depth (and deeper nitrogen foraging) could thus result in changes of tree-ring δ^15^N values over time.

Ontogenetic effects on tree-ring δ^15^N values should be excluded or accounted for when using tree-ring δ^15^N as a tool to assess changes in soil nitrogen cycling over time. Previous studies have done so statistically by including tree age as a factor (Hietz et al., 2011). But this is not an ideal way to disentangle the potential effects of tree development and of nitrogen deposition on tree-ring δ^15^N values, e.g., because ontogenetic changes in tree-ring δ^15^N could be non-linear and because of possible collinearity between ontogenetic effects and changes related to soil nitrogen cycling. The sampling design used in this study entirely accounts for ontogeny. However, it has the main disadvantage that it requires many trees as each tree is sampled only once. In addition, each sampled tree grows at a different location, with possibly specific soil properties and drainage characteristics. This increased spatial sampling may lead to a large background variance in δ^15^N values. Such a high variance further requires a large sample size for sufficient statistical power to detect temporal δ^15^N trends. Our power test revealed that for the study site in Cameroon and Thailand only changes leading to a total increase ≥1.5‰ from 1950 to 2010 could be detected with ≥90% certainty (Figure 5). Thus for small datasets and/or to detect small changes in δ^15^N this method is less suitable.

### Nitrogen translocation in sapwood

Another factor that could complicate the interpretation of trends in tree-ring δ^15^N values is nitrogen translocation. Nitrogen in wood has some radial mobility, meaning that it is not permanently fixed when wood is formed. There are many reports on translocation of nitrogen across ring boundaries from ^15^N labeling experiments (Nômmik, 1966; Mead and Preston, 1994; Colin-Belgrand et al., 1996; Schleppi et al., 1999; Elhani et al., 2003; Hart and Classen, 2003). Trees re-use nitrogen present in cambial cells during maturation and extract nitrogen before cell death during heartwood formation (Cowling and Merrill, 1966; Merrill and Cowling, 1966; Poulson et al., 1995). How the translocation of nitrogen affects δ^15^N values in tree rings is not entirely clear. As discussed above, we did not find a consistent correlation between tree-ring δ^15^N and nitrogen content.

To reduce translocation effects, an extraction procedure is commonly applied that removes a large fraction of the soluble (mobile) N compounds from tree-ring wood and retains structural N compounds in cell walls (Sheppard and Thompson, 2000). We performed a chemical extraction similar to the Sheppard/Thompson method on all samples. This extraction might partially account for the effects of nitrogen mobility, although several studies show that not all mobile N is removed with this method (see review in Gerhart and McLauchlan, 2014).

To completely avoid potential translocation effects on tree-ring δ^15^N values, one could focus δ^15^N analyses on heartwood only (Hietz et al., 2010). Heartwood does not contain living cells and therefore nitrogen cannot be translocated between growth rings. We did not exclude sapwood samples from the analyses presented here, because of the strong reduction in data points this would have caused in the radial sampling method (Figure 2). In our study species, the number of growth rings in sapwood was variable, but for most trees the sapwood area contained the last 15 to 25 growth rings (i.e., going back to 1995–1985). Therefore, excluding sapwood samples would not have allowed the method comparison presented. Furthermore, excluding sapwood will remove data from the last decades, the period during which changes in tropical nitrogen cycling are hypothesized to have occurred.

## Conclusions

We used a new sampling methodology that completely controls for potential ontogenetic effects on tree-ring δ^15^N values, but the method also increases the variance included in the data and therefore lowers the power to detect relatively small changes in δ^15^N. We did not find evidence for a long-term change of tree-ring δ^15^N values over time for the study site in Cameroon, a result that could have been caused by the low statistical power. For the studied trees from Thailand, our results support, to a limited extent, a previously reported increase in tree-ring δ^15^N values since ~1950 (Hietz et al., 2011). This change could reflect an increased nitrate leaching following anthropogenic nitrogen input. But again, a low statistical power hinders the interpretation of this result. For trees from Bolivia, a much higher statistical power allowed a more rigid conclusion. Here we show the presence of an ontogenetic trend in δ^15^N values, which disappeared when controlling for tree size. Taken together, our findings are not consistent with the idea that nitrogen cycles in tropical forests are generally shifting to more open systems.

Anthropogenic nitrogen deposition is expected to increase dramatically in the near future in most tropical forests due to increased land-use intensification, forest fragmentation, biomass burning, and fossil fuel emissions (Galloway, 2004; Galloway et al., 2008). Most tropical forests are hypothesized to be particularly sensitive to extra nitrogen inputs (Matson et al., 1999, 2002). Tree-ring δ^15^N values are a very useful and relatively cheap tool to study changes in nitrogen cycling in tropical forests. This study shows the presence of ontogenetic changes in tree-ring δ^15^N values. We therefore strongly recommend accounting for tree ontogeny before temporal trends in δ^15^N can be properly interpreted. Possible tree-size corrections include: analyzing only tree rings in the adult stage (e.g., when a tree reached the canopy); including tree-size as a covariate in statistical analyses (sensu Hietz et al., 2011) or, more strictly, by the fixed-diameter method outlined in this study.

### Conflict of interest statement

The authors declare that the research was conducted in the absence of any commercial or financial relationships that could be construed as a potential conflict of interest.
