# Supplementary material for: 15N in tree rings as a bio-indicator of changing nitrogen cycling in tropical forests: an evaluation at three sites using two sampling methods
Source: Front Plant Sci. 2015 Apr 9;6:229. doi: 10.3389/fpls.2015.00229 (PMC4390989; doi:10.3389/fpls.2015.00229)
Supplement: Supplementary file 1 [file Image1.PDF]

Supplementary Material

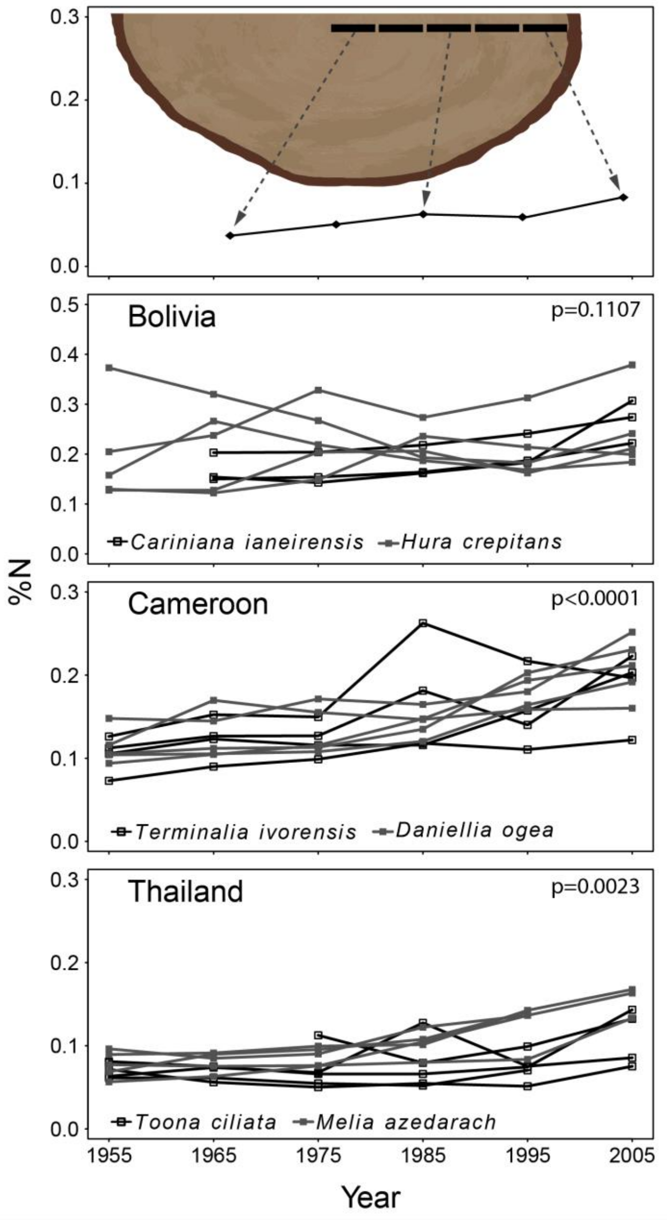

Figure S1. Tree-ring [N] values (as % of dry weight) in time using the method illustrated in the top panel (10-year bulk wood samples taken radially from 1950 to 2010). For each site, the two species were combined in a mixed-effect model, including ‘calendar year’ as a fixed factor and ‘individual tree’ as a random factor. The p-values for factor ‘calendar year’ are given in each panel.

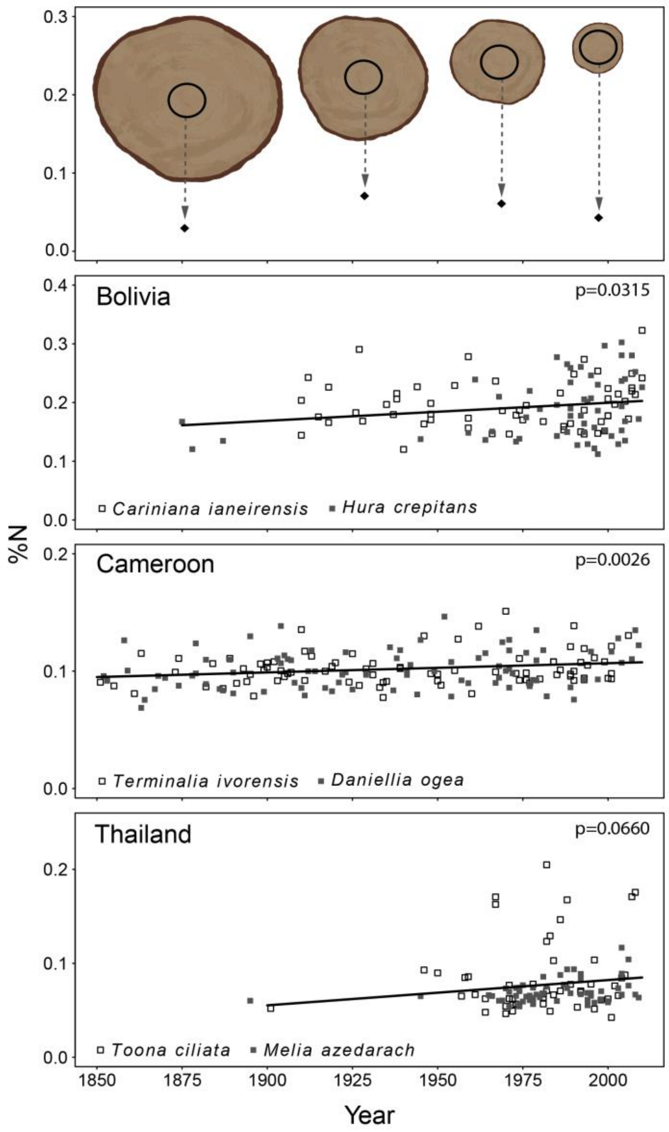

Figure S2. Tree-ring [N] values (as % of dry weight) in time using the fixed-diameter method illustrated in the top panel (10-year bulk wood samples around 20 cm dbh). For each site, the two species were combined in a mixed-effect model, including ‘calendar year’ as a fixed factor and ‘tree species’ as a random factor. Black lines represent per-site trends from the linear mixed effect models; p-values for factor ‘calendar year’ are given in each panel.
